# The performance of acute versus antecedent patient characteristics for 1-year mortality prediction during intensive care unit admission: a national cohort study

**DOI:** 10.1186/s13054-020-03017-y

**Published:** 2020-06-11

**Authors:** Monika C. Kerckhoffs, Sylvia Brinkman, Nicolet de Keizer, Ivo W. Soliman, Dylan W. de Lange, Johannes J. M. van Delden, Diederik van Dijk

**Affiliations:** 1 Department of Intensive Care Medicine, University Medical Center Utrecht, Utrecht University, Mail stop F06.149, P.O. Box 85500, 3508 GA Utrecht, The Netherlands; 2 National Intensive Care Evaluation (NICE) foundation, Amsterdam, The Netherlands; 3 Department of Medical Informatics, Amsterdam UMC, Amsterdam Public Health Research Institute, University of Amsterdam, Amsterdam, The Netherlands; 4 Department of Medical Humanities, Julius Center for Health Sciences and Primary Care, University Medical Center Utrecht, Utrecht University, Utrecht, The Netherlands

**Keywords:** Intensive care, Mortality, Prognosis, Advance care planning, Long-term outcomes, Critical illness

## Abstract

**Background:**

Multiple factors contribute to mortality after ICU, but it is unclear how the predictive value of these factors changes during ICU admission. We aimed to compare the changing performance over time of the acute illness component, antecedent patient characteristics, and ICU length of stay (LOS) in predicting 1-year mortality.

**Methods:**

In this retrospective observational cohort study, the discriminative value of four generalized mixed-effects models was compared for 1-year and hospital mortality. Among patients with increasing ICU LOS, the models included (a) acute illness factors and antecedent patient characteristics combined, (b) acute component only, (c) antecedent patient characteristics only, and (d) ICU LOS. For each analysis, discrimination was measured by area under the receiver operating characteristics curve (AUC), calculated using the bootstrap method. Statistical significance between the models was assessed using the DeLong method (*p* value < 0.05).

**Results:**

In 400,248 ICU patients observed, hospital mortality was 11.8% and 1-year mortality 21.8%. At ICU admission, the combined model predicted 1-year mortality with an AUC of 0.84 (95% CI 0.84–0.84). When analyzed separately, the acute component progressively lost predictive power. From an ICU admission of at least 3 days, antecedent characteristics significantly exceeded the predictive value of the acute component for 1-year mortality, AUC 0.68 (95% CI 0.68–0.69) versus 0.67 (95% CI 0.67–0.68) (*p* value < 0.001). For hospital mortality, antecedent characteristics outperformed the acute component from a LOS of at least 7 days, comprising 7.8% of patients and accounting for 52.4% of all bed days. ICU LOS predicted 1-year mortality with an AUC of 0.52 (95% CI 0.51–0.53) and hospital mortality with an AUC of 0.54 (95% CI 0.53–0.55) for patients with a LOS of at least 7 days.

**Conclusions:**

Comparing the predictive value of factors influencing 1-year mortality for patients with increasing ICU LOS, antecedent patient characteristics are more predictive than the acute component for patients with an ICU LOS of at least 3 days. For hospital mortality, antecedent patient characteristics outperform the acute component for patients with an ICU LOS of at least 7 days. After the first week of ICU admission, LOS itself is not predictive of hospital nor 1-year mortality.

## Background

Each year, millions of people receive intensive care unit (ICU) treatment. Most patients are admitted to the ICU for only a few days. Some patients, however, survive their initial acute illness but go on to experience persistent organ failure necessitating a prolonged stay in the ICU [1–4].

Prolonged treatment in the ICU may lead to increased suffering and high health care consumption [5, 6]. Moreover, it is known that patients with persistent critical illness have an increased hospital and 1-year mortality, with the highest mortality observed in the first months after discharge [3, 7–14].

Most prediction models used in the ICU predict hospital mortality [15–18]. Since most patients place emphasis on long-term outcomes when defining treatment goals, it is important to acknowledge long-term prognosis in order to make goal-concordant treatment decisions [10, 19].

Multiple factors contribute to the risk of mortality after ICU, for example, type of critical illness, physiologic derangement at admission, higher age, and frailty [11, 20–22]. Therefore, current prediction models use both acute illness variables and antecedent patient characteristics. However, it is unclear how each of these factors contributes to prognosis during the course of an ICU admission.

Recent studies described that for hospital mortality, the predictive value of antecedent patient characteristics (e.g., comorbidities, demographics) and acute characteristics (admission diagnosis and first day physiological derangements) change during ICU admission [3, 4]. After approximately 10 days in the ICU, antecedent patient characteristics start to outperform acute characteristics for the prediction of hospital mortality [3, 4]. There is growing evidence that also 1-year mortality and morbidity is mostly influenced by factors present before ICU admission, like frailty, co-morbidities, and healthcare utilization [8, 22–24]. The predictive value of length of ICU stay (LOS) for long-term outcome is not undisputed with studies showing heterogeneous results [15, 25–27].

This study aimed to compare the predictive value of acute characteristics on the one hand and antecedent patient characteristics on the other, for hospital mortality and 1-year mortality among patients with increasing ICU LOS. We hypothesized that the predictive value of the acute component would decrease as ICU LOS increases, while antecedent patient characteristics would have a more constant effect over time. In addition, we aimed to quantify the predictive value of ICU LOS itself for hospital mortality and 1-year mortality.

## Methods

### Aim

The aim of this study was to compare the predictive value for hospital and 1-year mortality of (a) acute admission characteristics and antecedent patient characteristics combined (regarded as current practice), with (b) acute component only, (c) antecedent patient characteristics only, and (d) ICU length of stay itself, among patients with increasing ICU LOS.

### Study design, setting, and data sources

This study was a retrospective observational cohort study, comprising all ICU patients included in the Dutch National Intensive Care Evaluation (NICE) registry between 1 January 2013 and 1 January 2018. The NICE registry routinely collects data of all Dutch ICU patients until hospital discharge, to monitor quality of ICUs by benchmarking [28].

Patient records in the NICE registry contain demographic, physiological, and diagnostic data of all consecutive ICU patients, including the Acute Physiology and Chronic Health Evaluation (APACHE) score, chronic co-morbidity, reason for ICU admission, and date of death when during hospital admission [29]. Date of death after hospital discharge was derived for each patient by linking patient data to a national administrative database of health insurance companies (Vektis, https://www.vektis.nl). Readmissions within the same hospitalization were only included for their first ICU admission. After discharge from the hospital, a re-admission to the ICU was included as a new admission. Patients with a missing length of ICU admission were excluded. Missing data on other included covariates was imputed by the median value of comparable groups of patients.

### Variables

The primary outcome measure was 1-year mortality. Secondary outcome measure was hospital mortality. We evaluated four groups of variables to predict 1-year mortality:

- Acute component and antecedent characteristics combined (see b and c)
- Acute component: diagnosis at ICU admission and acute (first 24 h) physiology [i.e., APACHE III Acute Physiology Score, APACHE IV reason for ICU admission categorized in 10 groups (cardiovascular, gastro-intestinal, genitourinary, hematological, metabolic, musculoskeletal, neurologic, respiratory, transplant, trauma), type of admission (medical, elective surgical, urgent surgical), lead time (calendar days, continuous variable), and source of ICU admission categorized in four groups (emergency room, operation room, general ward, other)] [17].
- Antecedent patient characteristics: demographics and chronic health components [i.e., gender, age, cirrhosis, neoplasm, hematological malignancy, immunocompromised, diabetes mellitus, chronic respiratory insufficiency, and renal insufficiency].
- Length of ICU stay divided into 24 h-periods

### Bias

Since data from all Dutch ICU patients are both in the NICE registry, as well as registered by Vektis, the risk of selection, information, and attrition bias is negligible.

### Statistical methods

Four different generalized mixed-effects models were developed. In all models, we corrected for the fact that patients in the same hospital are more similar than patients from different hospitals by including “hospital of admission” as random intercept. All continuous covariates were included as splines. The secondary outcome, hospital mortality, was assessed using the same mixed-effects models.

To assess the predictive performance of the model during ICU admission, the discrimination, expressed as the area under the receiver operating characteristics curve (AUC) of each model, was assessed for all patients with a length of ICU stay (LOS) of 0 days or longer, patients with a LOS of 1 day or longer, through patients with a LOS of 30 days or longer separately. These patient groups are not mutually exclusive, meaning that patients with a LOS of 4 days are also in the group of patients with a LOS of 1, 2, and 3 days. The AUC and 95% confidence interval (CI) were calculated using the bootstrap method [30]. In this method, the AUC of each generalized mixed-effects model was assessed in 500 bootstrap samples for each LOS patient group. Significant difference between the three models was tested using the DeLong method, with a *p* value of < 0.05 defined as being significantly different [31]. All statistical analyses were performed using R (Statistical Environment Package), version 3.6.0.

## Results

Between 1 January 2013 and 1 January 2018, 407,851 admissions were registered in the NICE registry. After exclusion of patients with missing length of stay (*n* = 7603, 1.9%), 400,248 admissions were included. There were 656 (0.2%) records with missing age and 3510 records (0.9%) with missing lead time. Missing age was imputed with the median age of patients of the same gender; missing lead time was imputed with the median value of patients with the same source of ICU admission. The reason for ICU admittance was medical in 48.5%, elective surgical in 38.9%, and 12.7% urgent surgical. Patient were more often male (59.5%) and 40.3% were mechanically ventilated at admission. Baseline characteristics are displayed in Table 1, detailed characteristics like co-morbidities are reported in Additional file 1. Table E1.

**Table 1:** Demographic characteristics of ICU patients admitted between 1 January 2013 and 1 January 2018

Length of ICU stay	≥ 0 days		≥ 2 days		≥ 7 days		≥ 14 days		≥ 21 days		≥ 28 days	
	Number	***%***	Number	***%***	Number	***%***	Number	***%***	Number	***%***	Number	***%***
Total	400.248	*100*	86.948	*21.7*	31.140	*7.8*	13.520	*3.4*	7.397	*1.8*	4.476	*1.1*
Male	238.306	*59.5*	52.521	*60.4*	19.308	*62*	8.566	*63.4*	4.786	*64.7*	2.938	*65.6*
Age*	66 (55–75)		67 (57–75)		66 (56–74)		66 (55–74)		66 (55–73)		66 (55–73)	
APACHE III score*	52 (37–71)		72 (55–93)		79 (61–99)		81 (63–101)		82 (64–102)		82 (63–102)	
Reason admission												
Medical	193.925	*48.5*	59.019	*67.9*	21.712	*69.7*	9.310	*68.9*	5.052	*68.3*	2.982	*66.6*
Urgent surgery	50.634	*12.7*	14.446	*16.6*	6.126	*19.7*	2.808	*20.8*	1.560	*21.1*	973	*21.7*
Elective surgery	155.689	*38.9*	13.483	*15.5*	3.302	*10.6*	1.402	*10.4*	785	*10.6*	521	*11.6*
Mech. Vent. at admission	161.419	*40.3*	48.917	*56.3*	19.576	*62.9*	8.757	*64.8*	4.862	*65.7*	2.971	*66.4*
Mech. Vent. in first 24 h	185.067	*46.2*	60.750	*69.9*	25.103	*80.6*	11.343	*83.9*	6.293	*85.1*	3.823	*85.4*
ICU mortality	32.152	*8.0*	13.132	*15.1*	6.112	*19.6*	2.750	*20.3*	1.390	*18.8*	795	*17.8*
Hospital mortality	46.186	*11.5*	18.709	*21.5*	8.357	*26.8*	3.694	*27.3*	1.866	*25.2*	1.076	*24.0*
Mortality 7 days after ICU admission	32.902	*8.2*	7.723	*8.9*	n.a	*n.a*	n.a	*n.a*	n.a	*n.a*	n.a	*n.a*
Mortality 28 days after ICU admission	50.981	*12.7*	18.832	*21.7*	7.337	*23.6*	2.432	*18.0*	655	*8.9*	n.a	*n.a*
Mortality 90 days after ICU admission	63.889	*16.0*	24.134	*27.8*	10.332	*33.2*	4.431	*32.8*	2.202	*29.8*	1.209	*27.0*
Mortality 180 days after ICU admission	73.152	*18.3*	26.805	*30.8*	11.411	*36.6*	5.008	*37.0*	2.591	*35.0*	1.510	*33.7*
Mortality 365 days after ICU admission	86.857	*21.7*	30.293	*34.8*	12.564	*40.3*	5.514	*40.8*	2.920	*39.5*	1.725	*38.5*

*Mech. Vent.* mechanical ventilation, *ICU* intensive care unit, *n.a*. not applicable

*for *Age/APACHE III* score, the median and interquartile ranges are reported

Of all 400,248 patients, 21.7% had a LOS of > 2 days, 7.8% of > 7 days, and 1.1% of patients were admitted for more than 28 days. ICU, hospital, and 1-year mortality for all patients treated in the ICU were 8.0%, 11.5%, and 21.7% respectively. For patients with an ICU stay of > 7 days, these values were 19.6%, 26.8%, and 40.3% respectively.

**Fig. 1:**
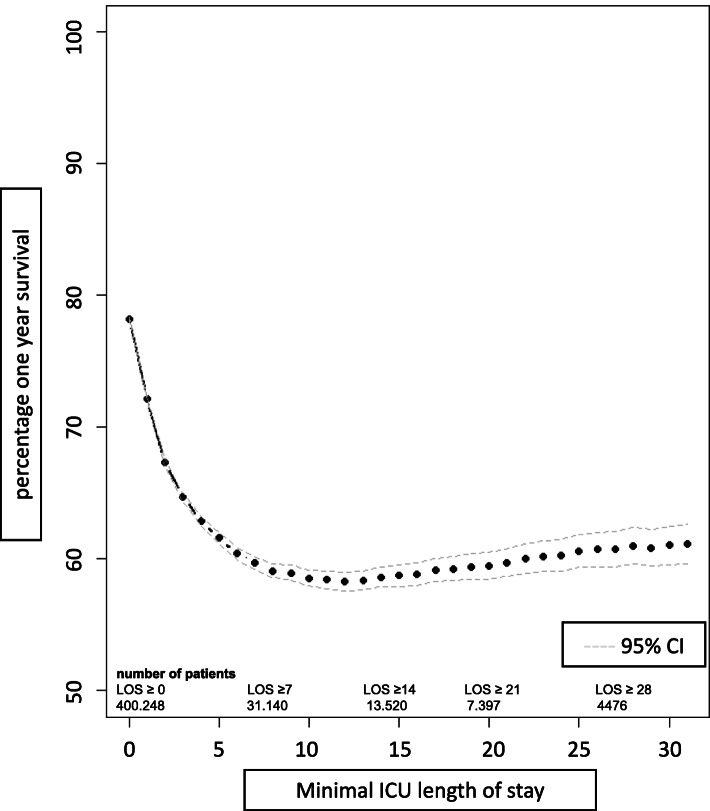
One year survival in relation to ICU length of stay. Dotted line illustrates percentage of patients that survived to 1 year in relation to the minimal ICU length of stay. Dashed lines indicate 95% confidence intervals. For different minimal durations of admission, the number of patients is shown in the bottom of the table. ICU, intensive care unit; LOS, length of stay in the intensive care unit

From all patients who died within the first year after ICU admission, 63.0% died after ICU discharge and in 46.8% after hospital discharge. Increasing length of ICU admission was associated with 1-year mortality during the first week. For patients with a LOS of > 7 days, 1-year mortality remained constant around 40%, also with increasing LOS (Fig. 1, Additional figure E1).

### Primary outcome: 1-year mortality

**Fig. 2:**
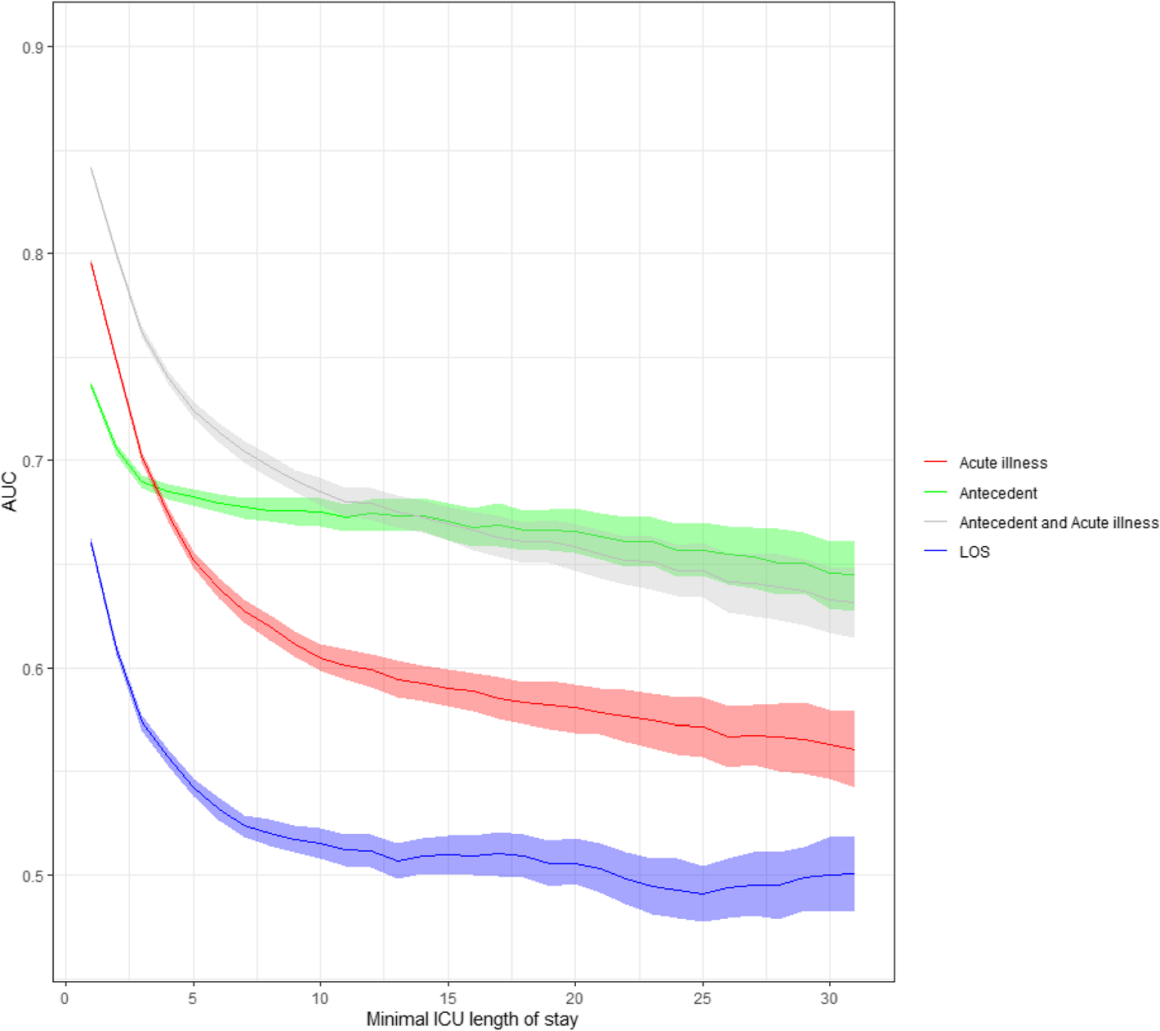
Predictive value of four different mixed models on 1-year mortality expressed by the AUC over time. Red, acute illness characteristics (diagnosis at ICU admission, acute physiology, type of admission, lead time, source of ICU admission). Green, antecedent characteristics (demographics, chronic health condition). Grey, combined model with acute illness characteristics and antecedent characteristics. Blue, duration of ICU stay in days. AUC, area under the receiver operating characteristics curve; LOS, length of stay; ICU, intensive care unit

In Fig. 2, the predictive value of acute and antecedent characteristics separately and combined and of LOS for 1-year mortality among patients with increasing LOS is illustrated. The model combining the acute component with antecedent characteristics had an AUC of 0.84 (95% CI 0.84–0.84) at admission. The AUC decreased over time to an AUC of 0.74 (95% CI 0.74–0.74) for patients with a LOS of at least 3 days and an AUC of 0.70 (95% CI 0.69–0.70) for patients with a LOS of at least 7 days. The acute component showed an AUC of 0.80 (95% CI 0.79–0.80) at admission, progressively decreasing for patients with a longer LOS to an AUC of 0.67 (95% CI 0.67–0.68) for patients with a LOS of at least 3 days and 0.62 (95% CI 0.61–0.63) for patients with a LOS of at least 7 days. The predictive value of the model using only antecedent patient characteristics showed an AUC of 0.74 (95% CI 0.73–0.74) at admission. The value declined and stabilized at an AUC of 0.68 (95% CI 0.68–0.69) for a LOS of at least 3 days and 0.68 (95% CI 0.67–0.68) when LOS was at least 7 days. Length of ICU stay had an AUC of 0.66 (95% CI 0.66–0.66) at admission, decreasing to an AUC of 0.56 (95% CI 0.55–0.56) for patients with a LOS of at least 3 days and 0.52 (95% CI 0.51–0.53) for at least 7 days.

Separating and comparing the acute component with the antecedent patient characteristics showed that for patients with a LOS of at least 3 days, the predictive value of antecedent characteristics on 1-year mortality significantly exceeded the acute component with an AUC of 0.68 (95% CI 0.68–0.69) versus AUC 0.67 (95% CI 0.67–0.68), *p* value < 0.001. From an ICU length of stay of 11 days or more, the predictive ability of the combined model and the antecedent patient characteristics are equal (AUC 0.68 (95% CI 0.67–0.69) versus AUC 0.67 (95% CI 0.67–0.68), *p* value 0.17.

### Secondary outcome: hospital mortality

**Fig. 3:**
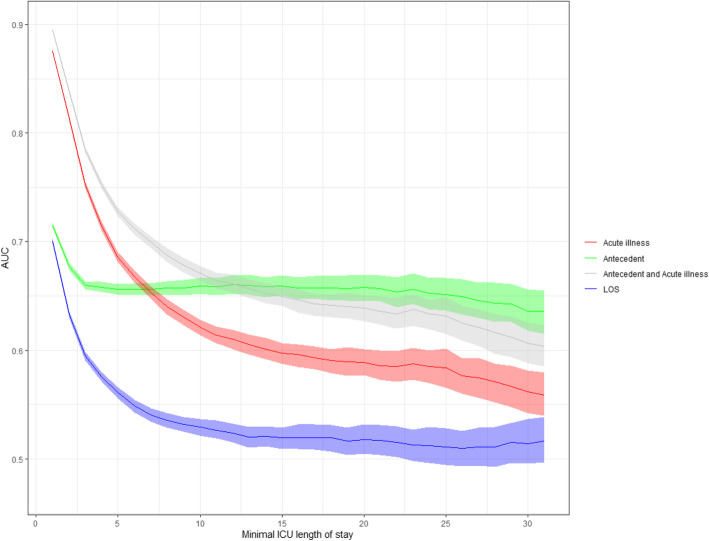
Predictive value of four different mixed models on hospital mortality expressed by the AUC over time. Red, acute illness characteristics (diagnosis at ICU admission, acute physiology, type of admission, lead time, source of ICU admission). Green, antecedent characteristics (demographics, chronic health condition). Grey, combined model with acute illness characteristics and antecedent characteristics. Blue, duration of ICU stay in days. AUC, area under the receiver operating characteristics curve; LOS, length of stay; ICU, intensive care unit

Figure 3 shows the predictive value of antecedent and acute factors separately or combined and of LOS for hospital mortality among patients with increasing LOS. The model combining the acute component with antecedent patient characteristics had an AUC of 0.89 (95% CI 0.89–0.90) at admission, compared to an AUC of 0.88 (95% CI 0.87–0.88) for the acute component and 0.71 (95% CI 0.71–0.72) for the antecedent patient characteristics.

The model using ICU length of stay to predict hospital mortality started with an AUC of 0.63 (95% CI 0.63–0.64) the first day after admission, decreasing to 0.54 (95% CI 0.53–0.55) for a LOS of at least 7 days.

Separating and comparing the acute component with the antecedent patient characteristics showed that for patients with a LOS of at least 7 days, the predictive value of the model using antecedent patient characteristics significantly exceeded the predictive ability of the acute component (AUC 0.66 (95% CI 0.65–0.66) versus 0.64 (95% CI 0.63–0.65) (*p* value < 0.001) for hospital mortality. This concerns 7.8% of all ICU patients, accounting for 52.4% of all bed days and 18.1% of the total hospital mortality.

For patients admitted for at least 10 days in the ICU, there is no significant difference between the predictive value of only the antecedent patient characteristics compared to the combined model (AUC of both models is 0.66, *p* value = 0.20) implying no impact of the acute component on hospital mortality.

## Discussion

In this national cohort study with over 400,000 ICU patients, we explored the predictive value for 1-year mortality of acute illness characteristics, antecedent patient characteristics, and length of stay in the ICU. We found that for patients with a LOS of at least 3 days, antecedent patient characteristics outperformed the acute component in predicting 1-year mortality. After the first week of ICU admission, length of ICU itself had no predictive value for long-term outcome.

### Timing

The moment when antecedent characteristics outperform acute illness characteristics in predicting hospital mortality has been described as the onset of persistent critical illness [3, 4]. In our large cohort, we found this moment at day 7, which is comparable to earlier studies. However, for the prediction of 1-year mortality, this moment was much earlier, namely after 3 days of admission. This difference in timing is likely to reflect the more pronounced effect of antecedent patient characteristics than of acute illness characteristics on survival beyond hospital discharge. Previous research has reported that acute illness influences short time survival, while mortality beyond 3 months was determined by age, co-morbidities, and frailty [8, 13, 20, 26, 32]. Comparable findings have been reported for resource use after hospital discharge and persistent physical symptoms, which are largely determined by factors present before ICU admission and seldom from ICU disease severity [23, 24].

In our study, antecedent patient characteristics showed a relatively constant predictive value for mortality after surviving the first days of ICU admission. Both for hospital mortality as well as for 1-year mortality, the models based on antecedent patient characteristics had a stable AUC over time. This effect has also been described for frailty, which affects short- and long-term mortality in a comparable manner [33, 34].

We found that beyond the first week, prolonged LOS cannot be used to predict mortality. Although the risk of 1-year mortality doubled in the first week in ICU (from 20 to 40%), the AUC curves were low and additional days in ICU after a LOS of 9 days did not contribute to the risk of mortality. Our Dutch data corresponds with that of a large Australian cohort, describing that treatment days beyond day 10 are unrelated to outcome in unselected ICU patients [26]. Although contra-intuitive, these findings stress that a prolonged ICU LOS should not immediately lead to pessimism regarding survival.

### Use in clinical practice

This study aimed to give insight in the specific contribution of the acute component and antecedent patient characteristics on 1-year mortality prediction during ICU admission. In current practice, both factors are combined when making outcome estimates. We showed that antecedent characteristics outperform the acute illness characteristics for patients with a LOS of at least 3 days. Moreover, the impact of admission diagnosis and severity, either high or low, diminished for both hospital mortality and 1-year mortality from a LOS of at least 10–11 days. However, all components showed relatively low AUC curves and should not be interpreted as prediction models. Since we currently lack reliable models able to predict survival beyond hospital discharge, acknowledging the change in importance over time of antecedent patient characteristics versus admission diagnosis and physiologic derangements is important. Our findings should be regarded as a strong argument to gather background information on patients early during ICU treatment. Especially in patients with a delayed recovery from their primary illness, pre-morbid health might be crucial in their chances of survival.

The strengths of our study include the use of a very large sample of unselected ICU patients, with the number of missing values of 0.9%, minimizing the risk of bias. This is also reflected by the small confidence intervals. However, there are also some limitations to be mentioned. Firstly, we expect that our findings regarding the predictive value of antecedent characteristics compared to acute critical illness are generalizable. However, due to differences in discharge and admission policy among countries, the moment when antecedent patient characteristics have better predictive value than acute illness characteristics might differ with respect to settings. In addition, we were not informed about the discharge locations. A small number of patients might be discharged to a hospice with death anticipated. Secondly, the covariates of the models may have affected the predictive value. For example, only the co-morbidities available in the NICE registry were used as antecedent patient characteristics. Although this is a pragmatic approach, it resembles the available information in daily practice; additional background information like frailty and functional dependency is likely to influence the predictive value. Furthermore, the elements of our acute illness model demonstrated limited predictive value for 1-year mortality, which could possibly be enhanced by improving the model by adding elements like vasopressor use or mechanical ventilation and thus affecting our results. Thirdly, we only have information about the mortality of ICU patients and not about functional capacity, cognitive, or mental disabilities following (prolonged) ICU treatment. Prolonged LOS could have adverse effects, not captured by mortality as a single outcome construct. Fourthly, mortality can be the result of treatment limitations, which introduced a risk of bias.

Future research could focus on combining the antecedent patient model with dynamic information during the ICU treatment, such as the development and severity of organ failure during admission. Such a dynamic model might be able to enhance the identification of patients who are (un) likely to have long-term survival.

## Conclusions

Models for prediction of ICU outcomes are typically based on a combination of antecedent patient characteristics with admission diagnosis and disease severity. Comparing the predictive value of these factors for 1-year mortality among patients with increasing ICU LOS, antecedent patient characteristics are more predictive than the acute component for patients with an ICU LOS of 3 days or more. For hospital mortality, antecedent patient characteristics outperform the acute component for patients with a LOS of at least 7 days in the ICU. After the first week of ICU admission, LOS itself is not predictive of hospital or 1-year mortality.

## Supplementary information

**Additional file 1: Table E1.** Detailed demographic characteristics. **Figure E1.** Percentage survival over time for groups with different ICU lengths of stay.

## Data Availability

The data that support the findings of this study are available from the NICE registry and Vektis, but restrictions apply to the availability of these data, which were used under license for the current study, and so are not publicly available. Data are however available from the authors upon reasonable request and with permission of both NICE and Vektis.

## Abbreviations

APACHE: Acute Physiology and Chronic Health Evaluation
CI: Confidence interval
ICU: Intensive care unit
LOS: Length of stay
NICE: National Intensive Care Evaluation
