# Supplementary material for: The performance of acute versus antecedent patient characteristics for 1-year mortality prediction during intensive care unit admission: a national cohort study
Source: Crit Care. 2020 Jun 11;24:330. doi: 10.1186/s13054-020-03017-y (PMC7291572; doi:10.1186/s13054-020-03017-y)
Supplement: Supplementary file 1 — Additional file 1: Table E1. Detailed demographic characteristics. Figure E1. Percentage survival over time for groups with different ICU lengths of stay. [file 13054_2020_3017_MOESM1_ESM.docx]

**Additional file** to The performance of acute versus antecedent patient characteristics for one-year mortality prediction during intensive care unit admission; a national cohort study.


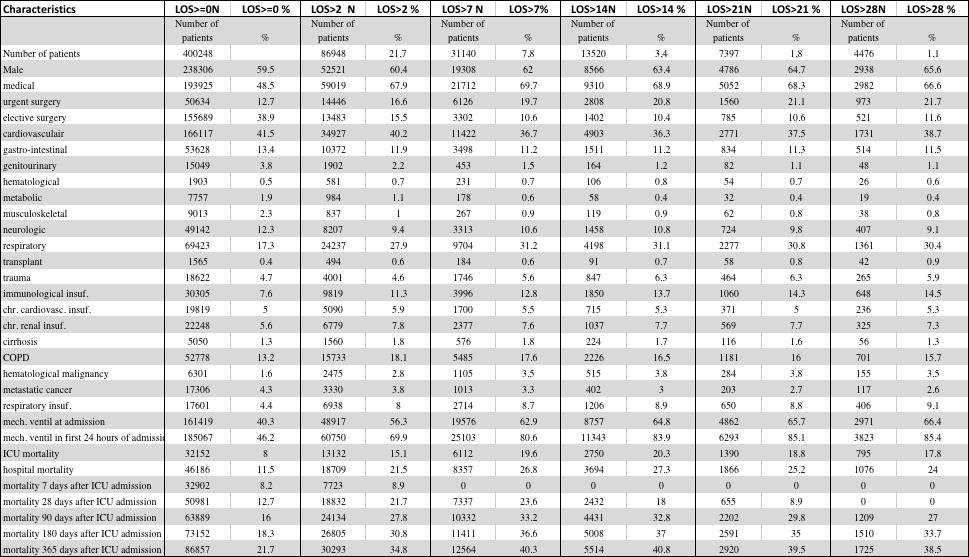


Table E1. Detailed demographic characteristics. *ICU* Intensive Care Unit, *COPD* Chronic Obstructive Pulmonary Disease

**Figure E1. Percentage survival over time for groups with different ICU lengths of stay**.


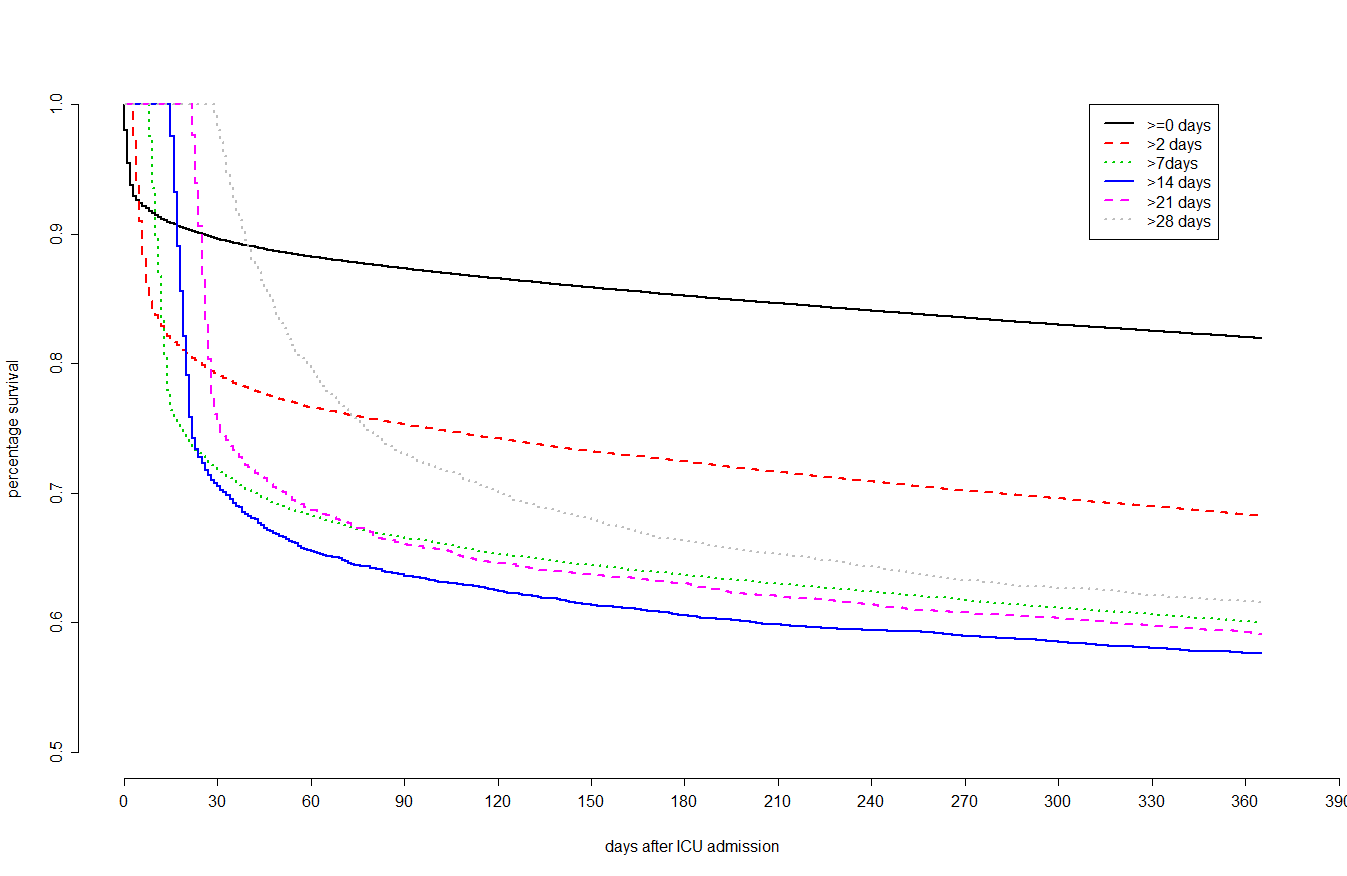


*ICU* intensive care unit
